# Effect of Rhizosphere Enzymes on Phytoremediation in PAH-Contaminated Soil Using Five Plant Species

**DOI:** 10.1371/journal.pone.0120369

**Published:** 2015-03-30

**Authors:** Rui Liu, Yuanyuan Dai, Libo Sun

**Affiliations:** 1 Key Laboratory of Pollution Ecology and Environmental Engineering, Institute of Applied Ecology, Chinese Academy of Sciences, Shenyang, Liaoning, China; 2 University of Chinese Academy of Sciences, Beijing, China; 3 Applied Engineering Department, Hangzhou Wanxiang Polytechnic, Hangzhou, Zhejiang, China; Leibniz-Institute of Vegetable and Ornamental Crops, GERMANY

## Abstract

A pot experiment was performed to study the effectiveness of remediation using different plant species and the enzyme response involved in remediating PAH-contaminated soil. The study indicated that species *Echinacea purpurea*, *Festuca arundinacea* Schred, Fire Phoenix (a combined *F*. *arundinacea*), and *Medicago sativa* L. possess the potential for remediation in PAH-contaminated soils. The study also determined that enzymatic reactions of polyphenol oxidase (except Fire Phoenix), dehydrogenase (except Fire Phoenix), and urease (except *Medicago sativa* L.) were more prominent over cultivation periods of 60d and 120d than 150d. Urease activity of the tested species exhibited prominently linear negative correlations with alkali-hydrolyzable nitrogen content after the tested plants were cultivated for 150d (R^2^ = 0.9592). The experiment also indicated that alkaline phosphatase activity in four of the five tested species (*Echinacea purpurea*, *Callistephus chinensis*, *Festuca arundinacea* Schred and Fire Phoenix) was inhibited during the cultivation process (at 60d and 120d). At the same time, the study determined that the linear relationship between alkaline phosphatase activity and effective phosphorus content in plant rhizosphere soil exhibited a negative correlation after a growing period of 120d (R^2^ = 0.665). Phytoremediation of organic contaminants in the soil was closely related to specific characteristics of particular plant species, and the catalyzed reactions were the result of the action of multiple enzymes in the plant rhizosphere soil.

## Introduction

Contamination by polycyclic aromatic hydrocarbons (PAHs) in the environment has become increasingly problematic with economic development, increasing energy consumption, and heavy emission of industrial “three wastes (waste water, waste gas, waste residue),”etc [1]. Many countries have placed PAHs onto blacklists of priority-monitored pollutants as PAHs have strong “three disease-causing” effects (carcinogenic, teratogenic and mutagenic), persistence in the environment, bio-accumulative characteristics and toxicity [2, 3]. Meanwhile, PAHs have high hydrophobicity and low bio-degradation properties, which can easily lead to their accumulation in the environment. Therefore, the PAHs are of wide concern in the current domestic and international environmental fields [4, 5], belonging to a class of serious problems that must be addressed by environmental research [6]. A significant amount of recent research has shown that PAH contamination can be measured in air, soil, water, and organisms to differing degrees. PAHs are often adsorbed onto soil particles due to poor water solubility and a high octanol-water partition coefficient. Soil becomes the primary carrier of these compounds and is able to remain undegraded for long periods of time [7, 8, 9].

Because the soil environment is characterized by multi-media, multi-interface, multi-component, heterogeneity and complexity, PAH contamination in soil is a notably complex environmental problem. This contamination has become a hot topic in current environmental science research into managing and restoring contaminated soils [10–13]. Currently, there are many techniques which can be used for restoring PAH-contaminated soil, including physical, chemical, and biological restoration techniques [7, 13, 14, 15]. Biological restoration techniques receive more attention every year due to their unique advantages of low cost, no secondary pollution, and large-area applicability, making the category one of the most promising for soil restoration [16–19]. Phytoremediation is the primary method among biological restoration techniques for controlling soil contamination in large areas, a technique in which contaminated soil is remediated by green plants. Phytoremediation of contaminated soil is achieved by the metabolic activities of the plants. Soil provides the moisture and nutrients essential for the plants to survive. Physical and chemical properties as well as biological environment (primarily microorganisms) have profound influences on plant absorption and pollutant transformation [20, 21]. Phytoremediation techniques have the advantage of low cost, no damage to the environment, and high acceptance among local residents as an emerging in-situ green restoration technique. These techniques have become a hot-spot for study and broad application across environmental science [21–24].

However, recent phytoremediation studies have primarily investigated screening efficient restoration plants. The mechanisms underlying phytoremediation are not well-understood, which has been a major obstacle in applying and practicing phytoremediation in large areas. Soil organic contaminants, such as PAHs, can be directly and indirectly removed here, “direct” refers to direct degradation of PAHs by enzyme systems, and “indirect” refers to improving living conditions for indigenous microorganisms, enhancing their activity and accelerating the degradation of PAHs [25, 26]. Enzymes are among the most important types of biological catalyst in soils. Enzymes can participate in many important biochemical processes in soil such as synthesis and decomposition of humus, hydrolysis of organic compounds and higher plant and microbial residues, conversion into more bioavailable forms, redox reactions, etc. [27]. Several studies have examined the role of enzymes in the soil contaminant restoration process in detail. Many enzymes are capable of transforming organic contaminants by catalyzing chemical reactions in soil. Khaziev (1980) [27] identified plant enzymes as the causative agents in the transformation of contaminants mixed with sediment and soil. The identified enzyme systems included dehalogenase, nitroreductase, peroxidase, and laccase. These findings suggest that plant enzymes may have significant spatial effects extending beyond the plant itself and temporal effects continuing after the plant dies [28].

Test plants in this experiment were chosen based upon experiments performed over the years, as selected from 14 plants. The ability to degrade PAHs in soil was studied further, including comparative analyses. At the same time, enzyme activities in rhizosphere soil were tested. The plants’ response and mechanisms in the phytoremediation of PAH-contaminated soils were examined. Five tested plants were combined in the experiment at the same time to analyze the degradation rate of PAHs in soil and assess their relative effects on soil, enzyme, plant (root), and influence factors. The experiment provides support for future studies of phytoremediation mechanisms.

## Materials and Methods

### Experimental design

The aged PAH-contaminated soil used for this study was obtained (sampled to a depth of 250 mm) from the Shengli Oil Field in Dongying City, Shandong Province, China, which was permitted by the Security and Environment Protection Department of the Shengli Oil Field. The soil analysis was performed by the Key Laboratory of Terrestrial Ecological Processes, Institute of Applied Ecology, Chinese Academy of Sciences, Shenyang, China. The contaminated soil was classified as a drained brown soil, pH 7.66, in which the carbon (C), phosphorus (P), nitrogen (N), and available P concentrations were 45.77, 0.65, 0.73 and 0.002 g kg^-1^, respectively. The uncontaminated reference soil samples were collected from Wanliutang Park, Shenyang, China. The reference soil sample was classified as a drained brown soil with a pH value 6.70, in which the concentrations of C, P, N and available P were 12.82, 0.44, 0.80 and 0.011 g kg^-1^, respectively. The range of the concentrations of PAHs in the contaminated soil collected was 228 to 398 mg kg^-1^. The collected soil samples were sieved through a 4.00 mm sieve to ensure homogeneity. According to the pre-test results, all of the plants tested could not grow in the aged PAHs contaminated soil directly. Through the addition of uncontaminated reference soil, the contaminated soil was diluted to the range of 34.17 to 122.46 mg kg^-1^ according to the experimental design. Its composition of ∑8PAHs was 1.17 to 3.05 mg kg^-1^ of fluoranthene, 0.84 to 3.42 mg kg^-1^ of pyrene, 2.71 to 13.77 mg kg^-1^ of benzo(a)anthracene, 0.67 to 5.02 mg kg^-1^ of chrysene, 3.97 to 25.95 mg kg^-1^ of benzo(b)fluoranthene, 0.94 to 12.67 mg kg^-1^ of benzo(k)fluoranthene, 6.06 to 26.76 mg kg^-1^ of benz(a)pyrene and 10.84 to 45.12 mg kg^-1^ of dibenzo(a, h)anthracene.

The five tested plants were #1(*Echinacea purpurea*), #2(*Callistephus chinensis*), #3(*Festuca arundinacea* Schred), #4(Fire Phoenix) and #5(*Medicago sativa* L.). These plants and their basic botanical characteristics are summarized in Table 1; the seeds of the five plants were purchased from the Kelaowu Seed Company, Beijing, China.

**Table 1:** Tested plants and their basic characteristics.

Tested number	General name	Scientific name	Family and genera	Basic characteristics
#1	Purple coneflower	*Echinacea purpurea* (L.) Moench	Asteraceae, *E*. *purpurea*	A perennial flowering plant averaging 1.2 m tall and 0.5 m wide at maturity.
#2	Aster Callistephus	*Callistephus chinensis* (L.) Nees	Asteraceae, *C*. *chinensis*	An annual plant, growing 20–80 cm tall with branched stems. The leaves are alternate, 4–8 cm long, ovate and coarsely toothed.
#3	Fawn	*Festuca arundinacea* Schreb	Poaceae, *F*. *arundinacea*	Evergreen, tuft-forming grass with a deep root system.
#4	Fire Phoenix		Poaceae, *F*. *arundinacea*	A type of combined *F*. *arundinacea*. Evergreen, tuft forming grass; deeply rooted specimen plants.
#5	Alfalfa	*Medicago sativa* Linn.	*Leguminosae*	A cool season perennial legume, with height up to 1 m and a deep root system sometimes stretching to more than 15 m.

The tested soil (2.5 kg) samples were added to 20 cm diameter pots. A disc of filter paper was placed at the bottom of each pot to prevent the dry soil from escaping through the drainage holes, and the pots were placed on saucers. The tested plant treatments (*n* = 9) were introduced to each pot at 15 days after germination of each seed.

Next, the plants studied in three replicates in the contaminated soil (∑8PAHs = 34.17–122.46 mg kg^-1^) were harvested after a 60-day, 120-day or 150-day cultivation period. The three replicates of the control (no plants and only soil) were also maintained simultaneously with the same contaminated soil. The soils in the control were processed identically at the time of plant watering, and all treatments were processed within 150 days. The plants were sown in a growth chamber with a cycle of 16 h/25°C in day and 8 h/15°C at night. The plants were watered every second day to maintain approximately 25% gravimetric water content. The experiment was performed from April 28th, 2009 to September 29th, 2009 and lasted for 150 days. The roots were shaken to dislodge loose soils and the attached rhizosphere soil samples were archived. The soil samples were transported to a laboratory on crushed ice in a cooler. The collected samples were stored at 4°C for the determination of PAHs and enzymes.

### PAH extraction and analysis

Integrated extraction and cleanup was performed by pressurized liquid extraction (PLE) with a Dionex ASE 200 accelerated solvent extractor. Briefly, 5 g of soil samples were ground in a mortar with approximately 5 g of diatomite. A 33 ml extraction cell was packed with two cellulose filters and 5 g of activated silica gel (for cleanup). The homogenized and dried sample was then transferred quantitatively to the extraction cell, and 200 μl of the surrogate standard solution were added directly on top of the sample and left undisturbed for 20 min to ensure full percolation throughout the sample. The remaining cell volume was filled with Ottawa sand (20–30 mm mesh) from AppliChem (Darmstadt, Germany) as an inert matrix. The Ottawa sand was pre-cleaned by being heated at 450°C overnight.

The PLE program was conducted as follows. A mixture of n-pentane and dichloromethane (9: 1 v/v) was used as a solvent at a pressure of 1.5 kPa and a temperature of 100°C; the oven heat ramptime was 7 min, and the program had two extraction cycles with a 5 min static time and a flush volume of 70%. The soil samples were extracted twice, and the extracts were combined and preconcentrated to < 2 mL under a gentle stream of nitrogen and subsequently transferred quantitatively to a 5-mL volumetric flask. Finally, 200 μl of the injection spike solution were added followed by the addition of isooctane up to the apparatus fill mark.

The concentrations of the eight target PAHs were determined using a gas chromatograph (Agilent 6890N) interfaced to an HP-5975B quadrupole mass spectrometer operating in the electron ionization mode (Agilent 5975B). The gas chromatograph was equipped with a 40-m ZB-5 capillary column with special dimensions (0.18 mm ID × 0.25 μm film thickness). Helium was used as the carrier gas, and the gas flow was 0.8 mL min^-1^. Aliquots of 1 μl were injected in split-less mode. The injector, ion source, and quadrupole temperatures were 325°C, 230°C, and 150°C, respectively. The oven program was: 35°C (held for 3 min), increased to 100°C (25°C min^-1^), then to 247°C (5°C min^-1^) and to 320°C at a rate of 3°C min^-1^ (held for 10.67 min) leading to a total analysis time of 70 min. Selected ion monitoring was used to analyze 21 m/z values in the range of m/z of 128 to 288 and divided into eight groups with 2 to 4 ions in each. Two six-point internal calibration curves (high and low concentrations) were used for quantification of the 8 PAHs. The concentrations were corrected for recovery.

### Enzyme activity

The polyphenol oxidase (PPO) activity was analyzed following the method of Guan (1986) [29]. 10 mL of 1.0% pyrogallol was added to a soil sample (1 g), and the reaction mixture was incubated at 30°C for 2 h. 4.0 ml of citric acid-phosphate buffer (pH 4.5) was added to the solution to stop the reaction, followed by the addition of 35 ml of ether. The mixture was extracted for 30 min. The colored ether with dissolved purple gallic prime was measured colorimetrically at a wavelength of 430 nm. Assays without soil and without pyrogallol were performed at the same time as controls.

The dehydrogenase (DHO) activity was analyzed using the protocol designed by Guan (1986) [29]. Three-hundredths of a gram of CaCO_3_ and 0.5 mL of 3% TTC were added to a soil sample (3 g) and the mixture was incubated at 37°C in the dark for 24 h after being mixed in the shaker. The mixture was extracted for 1 min after 5 mL of methanol was added. Next, the solution was filtered into a 50-mL volumetric flask using glass funnels, which were plugged with adsorbent cotton at the bottom of the funnels. The soils in the tubes were washed out into the funnels using methanol until no red color remained on the adsorbent cotton in the funnels. The samples were then measured colorimetrically at 485 nm after being diluted to 50 mL using methanol. Assays without CaCO_3_ and without TTC were performed at the same time as controls.

The urease (URE) activity was determined following the method of Guan (1986) [29]. Briefly, 0.5 ml toluene, 20 ml of pH 6.7 citrate buffer and 10 ml of 10% urea were added to 5.0 g soil, and then the mixture was incubated at 37°C for 24 h. The released ammonium was measured colorimetrically using Indophenol Blue Method at 578 nm. A control without urea was prepared with each sample.

The alkaline phosphatase (ALP) activity was measured using the method of Guan (1986) [29]. Soil (5 g) was incubated at 37°C with 20 ml 0.5% disodium phenylphosphate of borate buffer (pH 9.4) for 2 h. The phenol produced was extracted and oxidized by potassium hexacyanoferrate in alkaline solution. The oxidation products were determined using the 4-aminoantipyrine colorimetric method at 510 nm. Assays without soil and without disodiumphenyl phosphate were prepared at the same time as controls.

### Statistical methods

Statistical analysis was performed using Microsoft Excel XP and IBM SPSS 17.0. Sampling and chemical analyses were examined in triplicate to decrease the experimental errors and to increase the experimental reproducibility. The confidence of the data generated in the present investigations were analyzed by standard statistical methods to determine the mean values and standard deviation (SD). The values in the Figures were expressed as the mean ± SD of the three replicates. The differences among the treatments were analyzed by one-way ANOVA (LSD test).

## Results and Discussion

### Degradation Rate of PAHs

**Fig 1:**
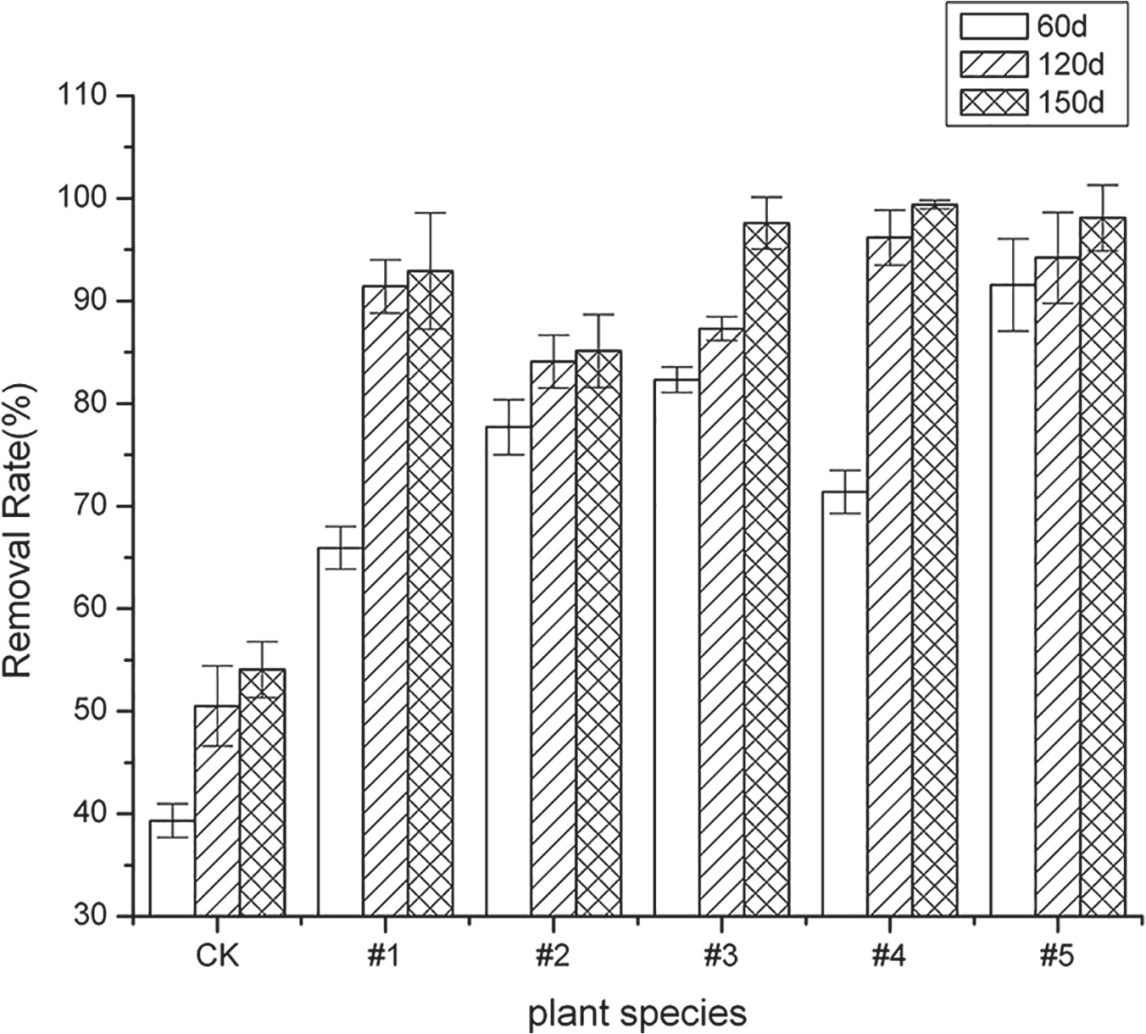
The removal rate of PAHs after 60 days, 120 days, and 150 days. The error bars indicate the standard deviation of the means (*n* = 3).

Fig 1 shows that the degradation rate of the five tested plants for ∑8PAHs (tetracyclic and pentacyclic) in rhizosphere soil had an upward trend with increasing cultivation time. The PAH degradation rate was significantly increased (*p* < 0.05) compared to the blank control, especially after 120 days of cultivation; the degradation rate of tested plants #1 and #4 increased notably from 77.67% to 84.09% and from 71.37% to 96.18%, respectively. It was also found that the degradation rate of PAHs in rhizosphere soils of the five tested plants was still somewhat increased after the plants were cultivated for 150d; the degradation rate of PAHs of four of the tested plants (#1(*Echinacea purpurea*), #3(*Festuca arundinacea* Schred), #4(Fire Phoenix) and #5(*Medicago sativa* L.)) exceeded 90% after being cultivated for 150d, reaching 92.91%, 97.59%, 99.4% and 98.11%, respectively. Total PAHs were respectively decreased from 122.46 to 8.68 mg kg^-1^, from 81.69 to 1.97 mg kg^-1^, from 78.62 to 0.47 mg kg^-1^, and from 38.63 to 0.73 mg kg^-1^. Summary analysis indicated that #1(*Echinacea purpurea*), #3(*Festuca arundinacea* Schred), #4(Fire Phoenix) and #5(*Medicago sativa* L.) possess better remediation potential than #2 (*Callistephus chinensis*) for PAH-contaminated soils and merit further study. This agreed with the results obtained by other studies [30, 31]. McCutcheon (2003) and Christensen-Kirsh (1996) studied several species of grass, such as *Agropyron smithii*, *Bouteloua gracilis*, *Cyanodon dactylon*, *Elymus Canadensis*, *Festuca arundinacea*, *Festuca rubra*, and *Melilotus officinalis* known to degrade PAHs. The results indicated that grasses and legumes enhance the removal of PAHs from contaminated soils. The plants included the legume alfalfa and three grasses—tall fescue, sudangrass, and switchgrass. Pyrene and anthracene were used as PAH contaminants. Planted soils had significantly lower concentrations of the PAHs than the unplanted soils, with 30 to 40% more degradation in the planted soils [32], and with 40 to 46% more degradation in the planted soil after a 150d-cultivation in the pot experiment.

### Polyphenol Oxidase

**Fig 2:**
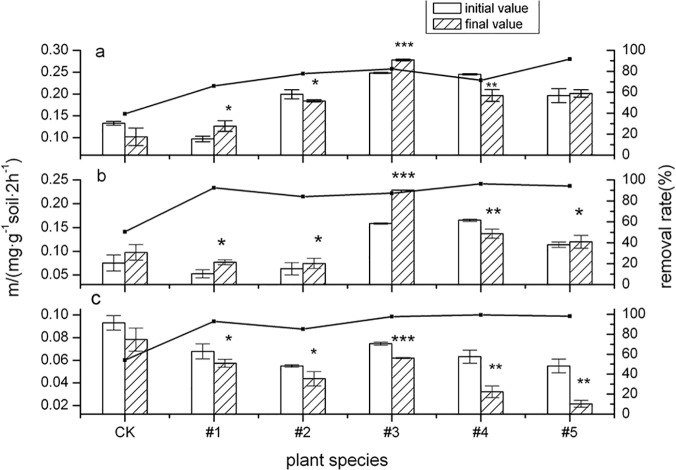
The activity of polyphenol oxidase (PPO) and its influence on ∑8PAHs degradation by five plant species over different time periods. The error bars indicate the standard deviation of the means (*n* = 3). Symbols *, ** and *** indicate significant differences at *p* levels < 0.1, 0.05 and 0.01, respectively. (Different letters denote different periods: 60 days (a), 120 days (b) and 150 days (c).)

Fig 2 shows the change in PPO activity of tested plants with increased cultivation time. It shows that the rhizosphere soil PPO activity of #1(*Echinacea purpurea*) (*p* < 0.1) and #3(*Festuca arundinacea* Schred) (*p* < 0.01) increased after 60d of cultivation. PPO activity in rhizosphere soil of the tested plants (except #4, Fire Phoenix) was prominently increased after being cultivated for 120d. The rate of activity increase was higher than during the first 60d of cultivation, which was closely related to the prominently increased PAH degradation rate in rhizosphere soil after being cultivated for 120d. Experiments have shown that the increase in PPO activity of plant rhizosphere soil is closely related to its efficiency in remediating PAHs in soil. Chen et al. (2004) [33] found that the activity of PPO was positively correlated with PAH concentration. PPO enzymatic reactions in the rhizosphere were most prominent at early stages of PAH pollution stress (60d and 120d). Good sources of nutrients were provided for soil biological communities with the degradation of PAHs in soil. Strong microbial flora was conducive to enhancing PPO activity. Meanwhile, PAH ring-opening reactions were catalyzed by increased activity of PPO. This made it easier to generate easily-degraded intermediates, thereby accelerating degradation, reducing the toxicity of the PAHs, and increasing the PAHs bioavailability [34]. Similar results were reported by Liu et al. (2004) [35], specifically that there were no significant differences in enzyme activity among the treatments within the first 60d. However, at later stages (60d-120d) the soil polyphenol oxidase activity (following addition of low and intermediate levels of B[a]P) was significantly higher than following the highest application rate (*p* < 0.05). Sun et al. (2010) [36] found that polyphenol oxidase activities in rhizosphere soil were significantly higher than in unplanted soil by sowing alfalfa. This suggests that the polyphenol oxidase mainly originated from the plant root.

Fig 2C shows that PPO activity in rhizosphere soil had a general downward trend after the initial logarithmic phase of activity increase. Analysis by comparison of Figs 1 and 2 indicates that PAH degradation rate slowly increased after a plant was cultivated for 150d. This phenomenon indicates that the soil biological systems adapted to PAH contamination by again forming PPO in the soil. This finding is also a major reason PPO helps promote phenol oxidation into quinones in the PAH degradation process. Quinones can be condensed with amino acids and peptides under suitable conditions, thereby forming initial humic acid molecules [37, 38].

Fig 2 shows that PPO activity for #4(Fire Phoenix) in the rhizosphere soil had a decreasing trend over the entire cultivation period. However, its degradation effect of ∑8PAHs in rhizosphere soil was still very good. The degradation rate of PAHs was the highest (99.40%) in tested plants possibly because of characteristics of the plant itself [39]. The experiment showed that PPO activity indicators in soil cannot be used as indicators during phytoremediation of PAH-contaminated soil. Plant remediation of organic contamination in the soil is closely related to characteristics of the plants themselves. Meanwhile, this remediation is promoted by the combined action of many enzymes and microorganisms in plant rhizosphere soils, and its study merits further detailed research.

### Dehydrogenase

**Fig 3:**
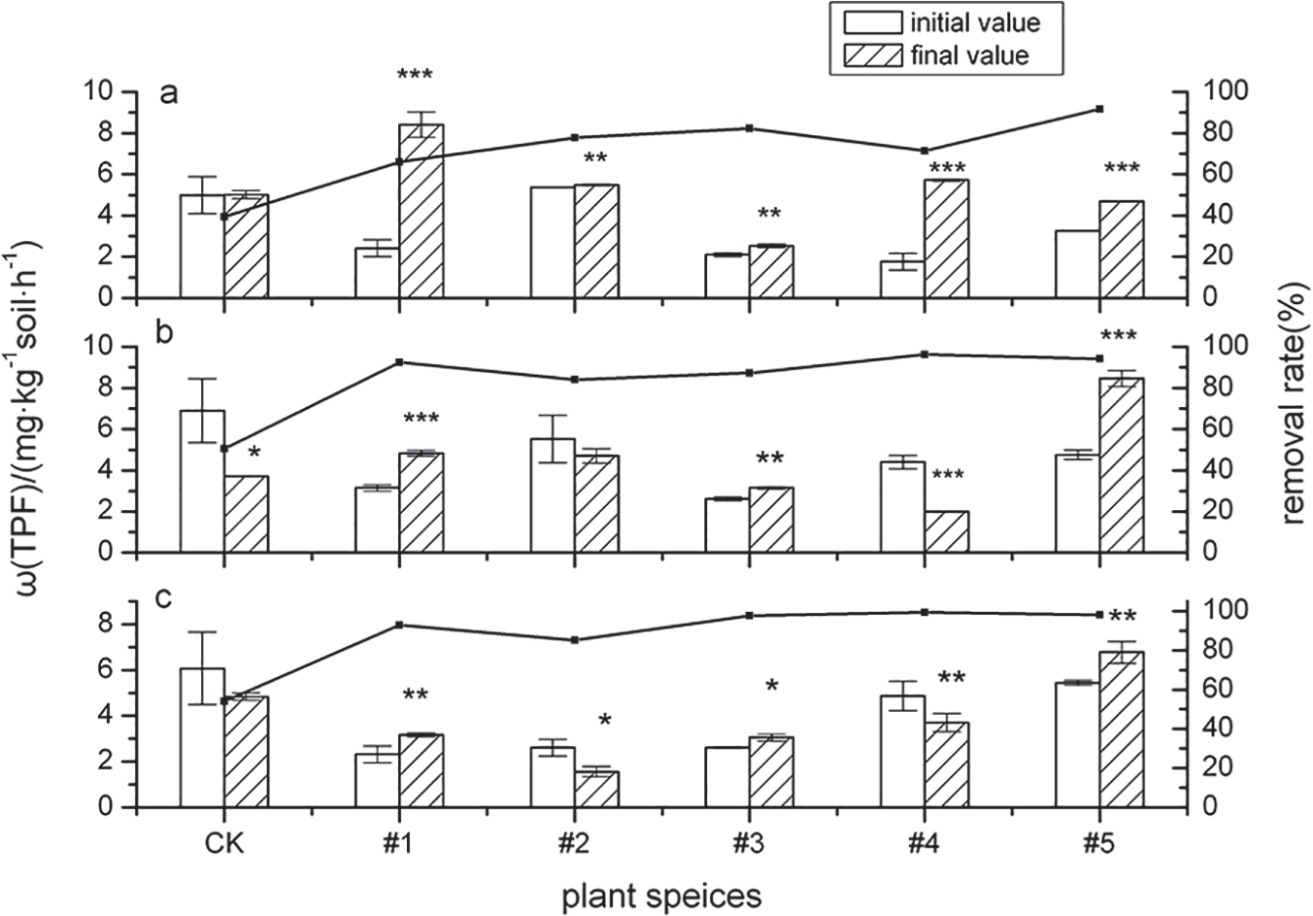
The activity of dehydrogenase (DHO) and its influence on ∑8PAHs degradation by five plant species over different time periods. The error bars indicate the standard deviation of the means (*n* = 3). Symbols *, ** and *** indicate significant differences at *p* levels < 0.1, 0.05 and 0.01, respectively. (Different letters denote different periods: 60 days (a), 120 days (b) and 150 days (c).)

Fig 3 shows the rhizosphere soil DHO activity change in different plant species and its influence on PAH degradation rate over different cultivation times. The plots show upward trends in DHO activity after the study plants were cultivated for 60d, notably higher than control group. DHO activity for #1(*Echinacea purpurea*) and #4(Fire Phoenix) increased by 2.49 times and 2.25 times, respectively. However, the DHO activity of #4(Fire Phoenix) decreased after being cultivated for 120d (*p* < 0.01) and 150d (*p* < 0.05). DHO activity in #1(*Echinacea purpurea*), #3(*Festuca arundinacea* Schred) and #5(*Medicago sativa* L.) continued to show an upward trend; #5(*Medicago sativa* L.) especially exhibited a significant increase of 77.86% after a 120-day growth period. These results were in agreement with those of Lee et al. (2008) [40] who also observed that DHO activity was much higher in planted than in unplanted soils during PAH treatment. DHO activity in planted soils increased with time in that study as well, whereas it remained relatively constant in unplanted soil. From the DHO activity data, the greatest stimulation of PAH-degrading microbes is expected in soils planted with *E*. *crus-galli* and *A*. *membranaceus*. Different observations were made by Cheema et al. (2010) [41], showing that the presence of PAHs stimulated DHO activity in the rhizosphere of all plant treatments except alfalfa. Experiments showed that the enzymatic dehydrogenation effect of plants on organic matter was greatly enhanced under PAH contamination stress; thereby effectively promoting and improving degradation of PAHs in rhizosphere soil [42]. PAH degradation rates in soils planted with #1(*Echinacea purpurea*), #3(*Festuca arundinacea* Schred), #4(Fire Phoenix) and #5(*Medicago sativa* L.) exceeded 90% after a 150-day growing period, also coinciding well with the observed higher DHO activity. Meanwhile, the PAH degradation rate in #2(*Callistephus chinensis*) rhizosphere soil only reached 84.09% 120 days after planting, which was related to the decrease of rhizosphere soil DHO activity. This conclusion was the same as that reported in previous studies; strong correlations between hydrocarbon removal and DHO activity are frequently observed [43, 44]. Meanwhile, Fig 3B and 3C also shows that DHO activity decreased for #4(Fire Phoenix) after growing at both 120 days (*p* < 0.01) and 150 days (*p* < 0.05); possibly because #4(Fire Phoenix) is itself a mixed grass, its performance characteristics (such as special plant root exudates, microbial community, etc.) are notably different from the other plants [39].

### Alkaline Phosphatase

**Fig 4:**
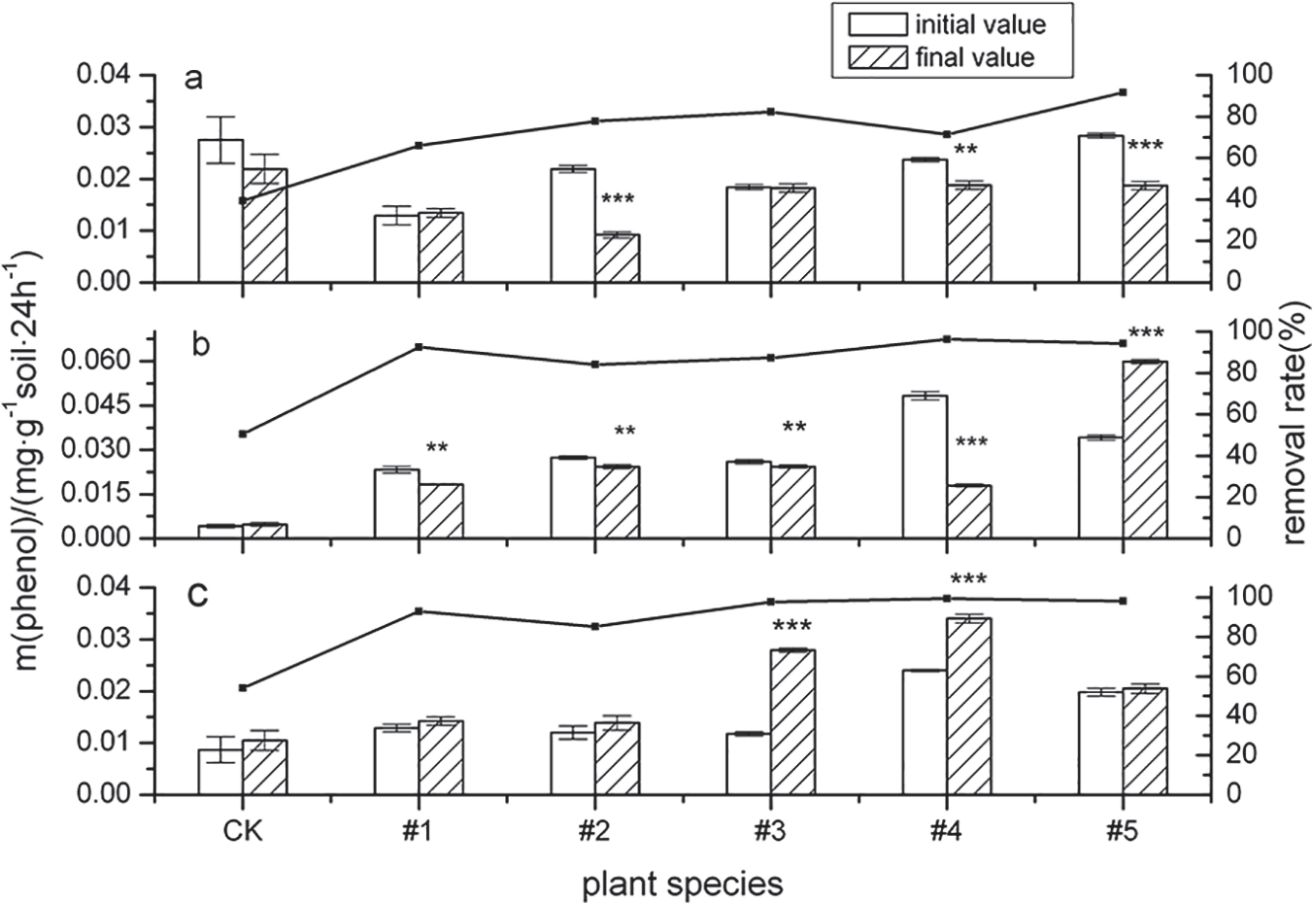
The activity of alkaline phosphatase (ALP) and its influence on ∑8PAHs degradation by five plant species over different time periods. The error bars indicate the standard deviation of the means (*n* = 3). Symbols *, ** and *** indicate significant differences at *p* levels < 0.1, 0.05 and 0.01, respectively. (Different letters denote different periods: 60 days (a), 120 days (b) and 150 days (c).)

Fig 4 depicts the rhizosphere soil alkaline phosphatase (ALP) activity changed after five tested plants were cultivated in PAH-contaminated soil for 60d, 120d and 150d. Fig 4A shows that ALP activity often decreased after the tested plants were cultivated for 60d, wherein #2(*Callistephus chinensis*) (*p* < 0.01) and #5(*Medicago sativa* L.) (*p* < 0.01) exhibited the most significant change, possibly because PAHs in contaminated soil destroyed the plant roots at the beginning of sowing, thereby reducing organic matter in the soil [45]. ALP activity was significantly reduced, and the inhibition effect lasted for a longer time. ALP activity in rhizosphere soil still decreased after #1(*Echinacea purpurea*), #2(*Callistephus chinensis*), #3(*Festuca arundinacea* Schred), and #4(Fire Phoenix) were cultivated for 120d. This conclusion was not consistent with the findings of previous studies. Andreoni et al. (2004) [46] observed a decrease in PAH levels with increased PHO activity in a Belgian soil.

Analyzing the characteristics of treated soil at 120 days after planting reveals a negative linear correlation between effective phosphorus content change in plant rhizosphere soil and change in phosphatase activity value (Y = -321.78 X + 86.761, R^2^ = 0.665). ALP activity in soil depends on the effective phosphorus content of plant roots, which is consistent with the finding [47] that ALP activity is negatively correlated with effective phosphorus content in soil. For #5(*Medicago sativa* L.), ALP activity increased prominently (*p* < 0.01) after being cultivated for 120d; perhaps because #5(*Medicago sativa* L.) belongs to a leguminous genus, there were rhizobia in the root systems which fix nitrogen in rhizosphere soil. ALP activity in soil is related to both effective phosphorus and total nitrogen content [47]. The mechanism thereof should be studied in greater depth.

### Urease

**Fig 5:**
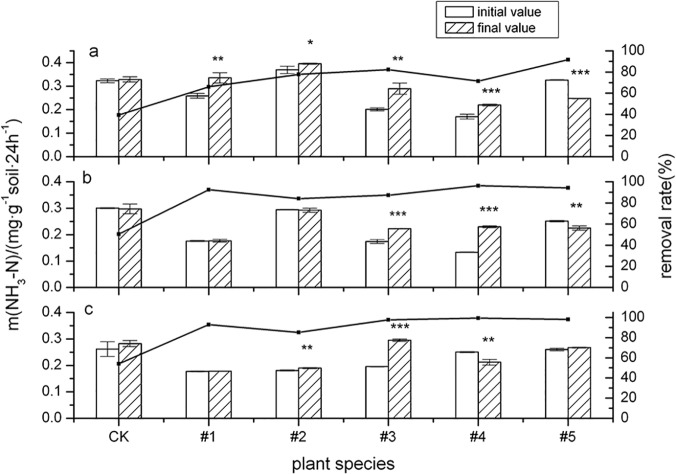
The activity of urease (URE) and its influence on ∑8PAHs degradation by five plant species over different time periods. The error bars indicate the standard deviation of the means (*n* = 3). Symbols *, ** and *** indicate significant differences at *p* levels < 0.1, 0.05 and 0.01, respectively. (Different letters denote different periods: 60 days (a), 120 days (b) and 150 days (c).)

Fig 5A and 5B shows that urease (URE) activity in the five tested plants (except #5, *Medicago sativa* L.) was generally enhanced after 60d and 120d of cultivation. These results indicate that PAHs in contaminated soil did not inhibit URE activity at the beginning of plant cultivation (60d and 120d). As various authors have reported [48, 49], such values may reflect the presence of a stabilized fraction of urease that is highly resistant to contaminants.

This experiment also showed that rhizosphere soil URE activity of #2(*Callistephus chinensis*) (*p* < 0.05) and #3(*Festuca arundinacea* Schred) (*p* < 0.01) was still elevated after a 150-day sowing. The URE activity change value for the five tested plants showed significant negative correlation with the alkali-hydrolyzable nitrogen change value (Y = -152.62 X—4.501, R^2^ = 0.9592). However, Khaziev (1980) [50] noted that URE activity in soil shows significant correlation inconsistency with nitrogen in soil. That study also found that URE was highly specialized, mainly acting on carbon and nitrogen bonds [50]. Increased URE activity is beneficial for plants, promoting hydrolysis of soil carbon and nitrogen bonds in soil PAHs, thereby assisting in degrading PAHs in rhizosphere soil. For #5(*Medicago sativa* L.), Fig 5 shows that URE activity generally decreased after sowing at both 60d (*p* < 0.01) and 120d (*p* < 0.05), possibly because this plant is a legume. Rhizobium was used for fixing nitrogen; therefore, URE activity was not increased by this type of stimulation in the early stages of planting. URE activity responded after a 150-day cultivation, the mechanism of which warrants further study.

Four of the tested plants (#1(*Echinacea purpurea*), #3(*Festuca arundinacea* Schred), #4(Fire Phoenix) and #5(*Medicago sativa* L.)) have excellent remediation potential on PAH-contaminated soil. Their degradation rates for ∑8PAHs (tetracyclic and pentacyclic) (34.17 mg kg^-1^–122.46 mg kg^-1^) can exceed 90% after a 150-day culture. Total PAHs were degraded from 122.46 to 8.68 mg kg^-1^, from 81.69 to 1.97 mg kg^-1^, from 78.62 to 0.47 mg kg^-1^, and from 38.63 to 0.73 mg kg^-1^, respectively. Enzyme response and influence mechanisms in plant rhizosphere soil during phytoremediation of PAH-contaminated soil can be assessed by examining the changes in oxidation reductase and hydrolase for the five tested plants. This experiment showed that rhizosphere soils can release oxidoreductase and hydrolase in the five tested plants after being cultivated for 60d, 120d and 150d. The enzyme activity of four of the five tested plants (except #4, Fire Phoenix) usually increased after both 60d and 120d for oxidoreductase (PPO and DHO); it decreased after being cultivated for 150d. This result illustrates that enzymatic reactions involving PPO and DHO are more prominent after 60d and 120d than 150d of cultivation and are inseparable from the prominently increased PAH degradation rate in soil during the same periods. However, the activity of these particular enzymes decreased after being cultivated for 150d. This suggests that enzyme activity equilibrates after 150d of cultivation. New soil enzymes are formed under PAH-contamination stress. Oxidoreductase, as key enzyme in PAHs degradation pathway, is critical in the PAHs degradation process. Only the DHO activity in #4(Fire Phoenix) increased at the beginning of cultivation (60d); PPO and DHO activity generally decreased in other cultivation stages, possibly because this plant is a mixed grass and so it may have different root exudates resulting from different microbial communities.

For hydrolases, ALP activity in rhizosphere soil of #1(*Echinacea purpurea*), #2(*Callistephus chinensis*), #3(*Festuca arundinacea* Schred), and #4(Fire Phoenix) generally decreased after being cultivated for 60d and 120d. This phenomenon also shows that ALP has a minimal role in promoting hydrolysis of PAHs during phytoremediation of PAH-contaminated soil. The ALP activity of #5 (*Medicago sativa* L.) showed an increase after a 120-day cultivation, possibly because it is a legume with roots that have specific microbial communities; the mechanism behind this effect should be further studied. The experiment also indicates that ALP activity in soil depends on phosphorus content of plant rhizosphere soil. A linear relationship between the ALP activity change and the effective phosphorus content change in rhizosphere soil showed negative correlation after 120 days of cultivation (Y = -321.78 X + 86.761, R^2^ = 0.665).

URE activity in rhizosphere soil generally increased after #1(*Echinacea purpurea*), #2(*Callistephus chinensis*), #3(*Festuca arundinacea* Schred) and #4(Fire Phoenix) were cultivated for 60d and 120d. This indicates that PAHs in contaminated soil did not inhibit URE activity at the beginning of plant cultivation (60d and 120d). URE activity of #5 (*Medicago sativa* L.) decreased during the cultivation process, possibly because this plant’s roots have rhizobia. The main function of hydrolase is to catalyze PAH hydrolysis. Soil provides energy for microorganisms generating URE during PAH degradation and URE activity is improved; the extent of this increase is related to the type of plant cultivated. Meanwhile, the experiment indicates that URE activity in the soil is related to alkali-hydrolyzable nitrogen in rhizosphere soil. The URE activity change of the five tested plants shows a prominent, negative linear correlation with change in the alkali-hydrolyzable nitrogen content (Y = -152.62 X—4.501, R^2^ = 0.9592) after the tested plants are cultivated for 150d.

## Conclusions

The study indicates that four of the tested species, #1(*Echinacea purpurea*), #3(*Festuca arundinacea* Schred), #4(Fire Phoenix) and #5(*Medicago sativa* L.) exhibit good potential to remediate PAH-contaminated soils. The study found that the enzymatic reactions of PPO (except for #4, Fire Phoenix), DHO (again, except for #4), and URE (except #5, *Medicago sativa* L.) were more prominent at cultivation periods of 60d and 120d than 150d, a trend that is inseparable from the prominently increased PAH degradation rate in soil during that period. Meanwhile, ALP activity in four of five tested plants (#1–#4) was inhibited during the cultivation process (at 60d and 120d). This result indicates that ALP has a minimal role in promoting hydrolysis of PAHs during the phytoremediation of PAH-contaminated soil. Phytoremediation of organic pollutants in soils is closely influenced by characteristics particular to the type of plant; meanwhile phytoremediation is also promoted by comprehensive catalysis of many enzymes (including oxidoreductase and hydrolases) in plant rhizosphere soil. These complexities would benefit from detailed characterization by further studies, which will likely prove a fruitful field of inquiry.
